# Incorporating the pedigree information in multi-environment trial analyses for improving common vetch

**DOI:** 10.3389/fpls.2023.1166133

**Published:** 2023-08-16

**Authors:** Isabel Munoz Santa, Stuart Nagel, Julian Daniel Taylor

**Affiliations:** ^1^ School of Agriculture, Food and Wine, The University of Adelaide, Adelaide, SA, Australia; ^2^ Department of Statistics and Operations Research, University of Valencia, Valencia, Spain; ^3^ South Australian Research and Development Institute, Adelaide, SA, Australia

**Keywords:** common vetch, factor analytic model, factor analytic selection tools (FAST), genetic by environment interaction, multi-environment trial, pedigree

## Abstract

Common vetch is one of the most profitable forage legumes due to its versatility in end-use which includes grain, hay, green manure, and silage. Furthermore, common vetch is one of the best crops to rotate with cereals as it can increase soil fertility which results in higher yield in cereal crops. The National Vetch Breeding Program located in South Australia is focused on developing new vetch varieties with higher grain and dry matter yields, better resistance to major diseases, and wider adaptability to Australian cropping environments. As part of this program, a study was conducted with 35 field trials from 2015 to 2021 in South Australia, Western Australia, Victoria, and New South Wales with the objective of determining the best parents for future crosses and the vetch lines with highest commercial value in terms of grain yield production. A total of 392 varieties were evaluated. The individual field trials were combined in a multi-environment trial data, where each trial is identified as an environment. Multiplicative mixed models were used to analyze the data and a factor analytic approach to model the genetic by environment interaction effects. The pedigree of the lines was then assembled and incorporated into the analysis. This approach allowed to partition the total effects into additive and non-additive components. The total and additive genetic effects were inspected across and within environments for broad and specific selections of the lines with the best commercial value and the best parents. Summary measures of overall performance and stability were used to aid with selection of parents. To the best of our knowledge, this is the first study which used the pedigree information to breed common vetch. In this paper, the application of this statistical methodology has been successfully implemented with the inclusion of the pedigree improving the fit of the models to the data with most of the total genetic variation explained by the additive heritable component. The results of this study have shown the importance of including the pedigree information for common vetch breeding programs and have improved the ability of breeders to select superior commercial lines and parents.

## 1 Introduction

Common vetch (*Vicia sativa L.)* is a self-pollinated legume specie of the Fabaceae family. It is the most widespread and economically important of the *Vicia sativa* aggregate (Mihailovich et al., 2005). In fact, common vetch is an extremely important annual forage and grain legume, particularly in low rainfall marginal farming areas, but also in high value cropping systems. Its value is related to its genuine multi-purpose nature. Farmers can decide on using vetch for silage, hay, grain, grazing or green manure (Matić et al., 2005). Plant biomass and grains can be used as valuable feed for ruminants, and the latter can be also incorporated in the diets of other animals up to a maximum of 22%, 15%, 10% and 10% for pigs, layer hens, rabbits, and broilers, respectively (Huang et al., 2017). The cultivation of vetch also provides multiple on-farm benefits. For example, it is beneficial for controlling grass weeds and it is used as a disease break of cereals (Pala et al., 2000; Mariotti et al., 2006; Vasilakoglou et al., 2008). Additionally, its symbiosis with rhizobia ensures the fixing of atmospheric nitrogen in the soil resulting in a reduction in the applications of nitrogen fertilizers, higher soil fertility (Unkovich et al., 1997; Zhang et al., 2021), and subsequent increased yield in crops sown after vetch has been harvested or incorporated (Dalias and Neocleous, 2017; Wang et al., 2021).

Vetches originated in Southern Europe or South West Asia (Maxted and Bennett, 2001; Potokina et al., 2002) but their adaptability to diverse soil types, and tolerance to cold, heat and dry conditions (Huang et al., 2017) have made vetches a widely adoptable crop in many other arable areas and it is now grown on every arable continent. Currently, the top three vetch producers of the world are Ethiopia, Russia, and Mexico^1^. Australia is also one of the main vetch producers, being eighth in ranking^1^. Australia has an export-oriented agriculture which plays an important role in the response to the increasing food demand, 50% by 2050 in the case of protein (Henchion et al., 2017), caused by population growth. For instance, in 2014, from the $53 billion production of agricultural commodities, $41 billion were exported, and the value of agricultural exports is forecasted to double (Daly et al., 2015) by 2050 when the global population is projected to be 9.7 billion (Lal, 2016), and 37.6 million in the Australian territory (Gao and Bryan, 2017). Therefore, increasing the productivity of the Australian agricultural systems while preserving ecosystem services is crucial to meet domestic and global market demands. In this context, vetch represents an attractive and sustainable rotation crop option to feed livestock while enhancing soil fertility and productivity.

Since 1993, the National Vetch Breeding Program (NVBP) at the South Australian Research and Development Institute (SARDI) has worked towards increasing farm productivity by developing vetch varieties with higher grain and dry matter yields, better resistance to major diseases, and wider adaptability to Australian cropping environments (Matić et al., 2007). As part of this program, a series of 35 field trials were conducted from 2015 to 2021 to evaluate grain production of a total of 392 common vetch lines in different growing environments across South Australia (SA), Western Australia (WA), Victoria (VIC), and New South Wales (NSW). Data from individual trials are frequently combined in what is called a multi-environment trial (MET) data set (Patterson et al., 1977; Smith et al., 2001; Welham et al., 2010; Fanning et al., 2018; Sissons et al., 2020). The environments are defined as the combinations of years and locations which provide the different growing conditions associated to different meteorological and agro-ecological factors. This type of MET data is usually analyzed using the factor analytic (FA) approach of Smith et al. (2001). This method handles unbalanced data (i.e. not all the varieties appearing in all the environments) and enables modelling the heterogeneity of errors between trials, the different sources of spatial variability within each trial (Gilmour et al., 1997), and the heterogeneity of variances-covariances of genetic by environment (G x E) interaction effects.

In many instances, a pedigree of all the lines evaluated in the MET is available and can be assembled with the aim to be incorporated into the analysis of the data. By assembling the pedigree of the lines, complex relationships from related individuals can be taken into account to predict the additive genetic effects, the so-called breeding values, through the inverse of the additive relationship matrix in the linear mixed model equations (Henderson, 1976; Meuwissen and Luo, 1992). This was first used in animal breeding studies where the pedigree has been widely applied. Some examples are Quaas and Pollak (1980) who discussed the methodology in Henderson (1976) for beef cattle testing programs, Brown et al. (2000) for a multi-trait scenario in sheep, or Bergsma et al. (2008) in a study of the social effects in the heritable variance for domestic pigs. Current studies (Melnikova et al., 2021; Zhang et al., 2022; Kaseja et al., 2023) combine the classical pedigree relationships with those obtained from genetic marker data to obtain more accurate predictions of breeding values (Aguilar et al., 2010).

In plant breeding studies, the utilization of the pedigree was motivated by its applications in animal breeding. Some of the first examples can be found in Panter and Allen (1995) for predicting the rankings of future soybean crosses for yield performance, Durel et al. (1998) for a multi-trait apple breeding program, or Dutkowski et al. (2002) who modelled the spatial variation in forest genetic trials to improve predictions of breeding values. All these studies estimated the genetic additive effects and disregarded the non-additive effects. As crops can be replicated, Oakey et al. (2006, 2007, 2008) further partitioned the total genetic effects into additive and non-additive effects and extended the MET models in Smith et al. (2001) to include the pedigree information. Comparisons of the standard analyses, where lines were assumed independent, with those which accounted for the pedigree relationships were evaluated in Oakey (2008). In this dissertation, nine different scenarios were simulated with different levels of additive variance as percentage of the total genetic variance, and genetic variance as percentage of total variance. In all the cases, incorporating the pedigree information resulted in a reduction of the mean square error of prediction for the total and additive genetic effects. Since the pedigree MET models developed in Oakey et al. (2007), this technique has been widely used in plant breeding studies. Mathews et al. (2007) applied it to study the grain yield adaptation of representative Australian and CIMMYT spring bread wheat across a large sample (106 trials) of Australian and international wheat production environments. The pedigree MET models provided a better fit to the data and allowed to investigate the different patterns in the additive and non-additive genetic by environment interaction effects. The superiority of the fit of the models which incorporated pedigree information was also demonstrated in the analysis of yield and oil content data in a series of canola trials in Australia (Beeck et al., 2010) and in series of potato field trials to assess powdery scab resistance in New Zealand (Paget et al., 2014). Other examples of the applications of this methodology can be found in Real et al. (2014) in the breeding of the perennial legume *Bituminaria bituminosa*, Cullis et al. (2014) for *Pinus radiata*, Asfaw et al. (2021) for white Guinea yam, and Cowling et al. (2023) for canola in Australia and Canada.

Despite the advantages of conducting a pedigree MET analysis and its wide application in plant breeding programs, to the best of the authors’ knowledge, this technique has not been used in any breeding program to improve common vetch. In this study, we aim to evaluate the inclusion of the pedigree information in the MET of NVBP common vetch trials to provide an improved ability to select superior commercial lines and parents for future crosses in terms of their grain yield performance.

## 2 Materials and methods

### 2.1 Experimental sites

The field trials evaluated in this study were in typical Australian farming regions for common vetch. The NVBP selected the trial locations to represent the areas where vetch is currently grown or can be adopted as an important crop in future. Trials were conducted in VIC, SA, NSW, and WA from 2015 to 2021 and represented different environments. Trials were sown with a target of 60 plants/m^2^ with conventional tillage equipment. Experimental plots sown by the NVBP were all 10 m in length by 1.36 m wide. The ones sown by contractors differed in dimensions but maintained the target of 60 plants/m^2^. Sowing times differed between locations and years and were targeted after the opening rains in April, the end of April to the end of May being the ideal time for sowing vetch in southern Australia. Each trial was maintained in accordance with local farmer practices, depending upon soil type, and climatic conditions. Trials in SA did not receive fertilizer, inoculant, or fungicides. The WA trial had 100kg/ha of Macro Pro Extra fertilizer applied pre-sowing. Those in NSW and the one in Birchip (VIC) had 75kg/ha of MAP and 60kg/ha of Granulock SZ Blend, respectively, applied at sowing. Due to the varying soil types, different combinations of chemicals were used for pest control. All trials received post-sowing pre-emergent herbicide applications to control broadleaved weeds, with in season application of selective grass herbicides used as necessary. Due to the nature of plot trials, insecticides were used in a preventative manner to provide control of red legged earth mite, lucerne flea, aphids, and native budworm at different times during the season.

Trials were taken through for grain production and harvested, at maturity, using modern plot harvesters, with each plot weighed to produce final grain yields converted to t/ha for analysis.

### 2.2 Experiment establishment and design

**Figure 1:**
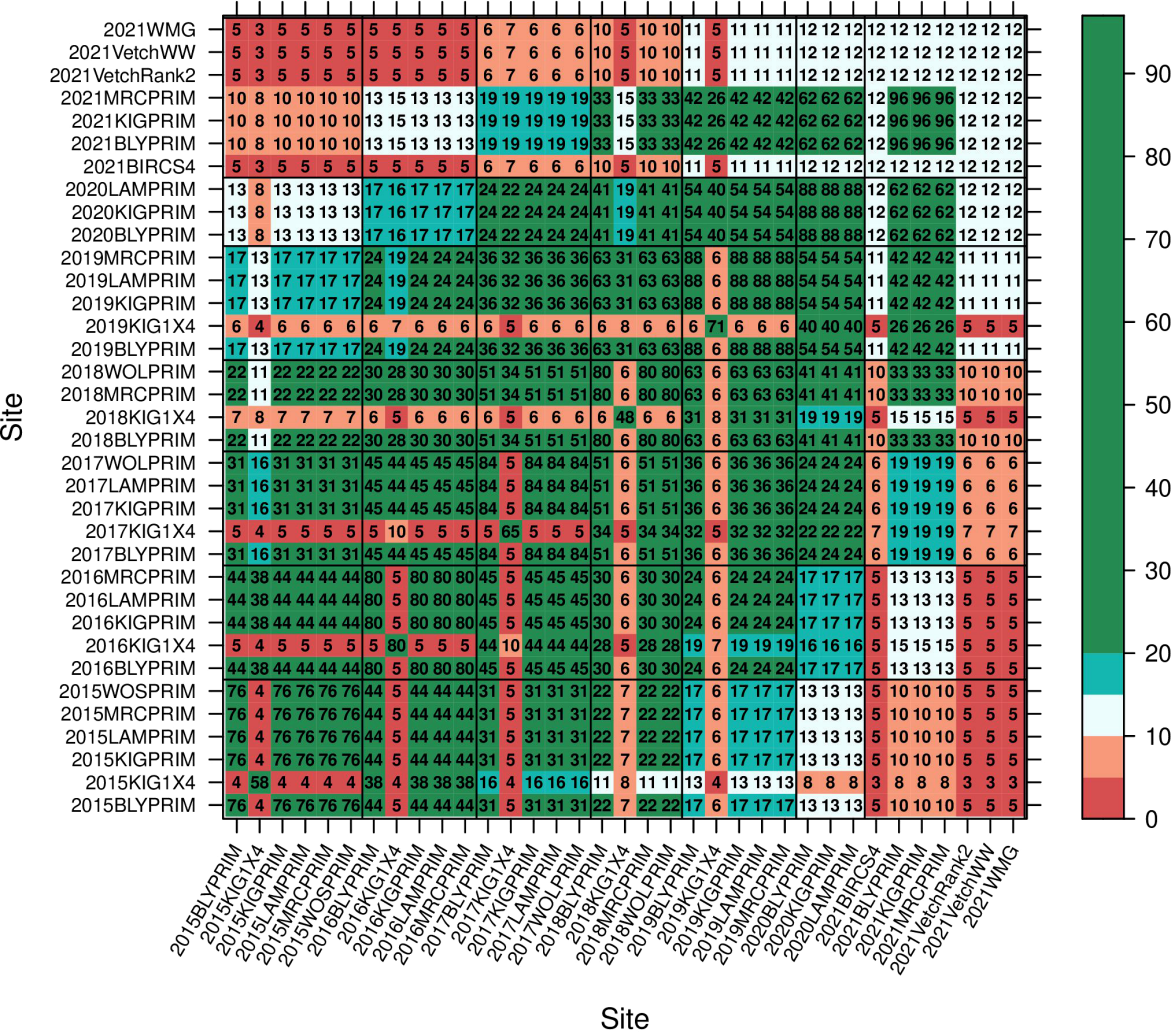
Concurrence of lines between trials. Numbers in the diagonal provide the number of lines per trial. Each number in the off-diagonals indicates the number of lines in common between pairs of trials which can go from low number of lines in common (0 -10 red), reasonable number (10 – 20 grey/blue) to more than 20 lines in common which is considered high concurrence (green).

The series of field experiments consisted of 35 trials (**Table 1**) with a total of 392 lines. Trials were named with the year followed by an acronym of the site. The MET data was highly unbalanced with 29% of the lines appearing in only one trial, 51%, 16%, 3% and 1% in 2 to 10, 11 to 20, 21 to 30 and 31 to 35 trials, respectively. The number of lines per trial is given in the diagonal elements of the concurrence matrix plotted in **Figure 1**. Most trials presented reasonable or high levels of line concurrence between pairs of trials (off diagonal numbers in **Figure 1**) allowing to combine the data across trials for the MET analysis (Section 2.4). All trials were laid out in a rectangular array of field plots indexed by rows and columns. Trial rows ranged from 6 to 96 and columns from 3 to 6. Trials were designed in a randomized complete block design with most trials presenting four blocks except the ones located in Minnipa (SA) and the interstate trials in 2021 which had three. The 2021VetchWW trial had blocks running in two directions.

**Table 1:** Description of trials evaluated in the study.

ID	Trial	Year	Nearest Town	State	Latitude/ Longitude	Mean rainfall mm	Annual rainfall mm	Soil type
1	WMG	2021	Dandargan	WA	30°20’41.3”S 115°32’47.4”E	537	404	deep red sand
2	WW	2021	Wagga Wagga	NSW	35°03’08.2”S 147°21’02.1”E	527	506	red brown earth/ kandersol
3	RANK 2	2021	Rankine Springs	NSW	33°59’29.3”S 146°08’40.1”E	416	661	red clay loam
4	MRCPRIM	2021	Minnipa	SA	32°50’02.5”S 135°09’03.6”E	327	358	calcareous sandy loam
5		2019	Minnipa	SA	same as 4	327	235	same as 4
6		2018	Minnipa	SA	same as 4	327	244	same as 4
7		2016	Minnipa	SA	same as 4	327	378	same as 4
8		2015	Minnipa	SA	same as 4	327	314	same as 4
9	KIGPRIM	2021	Roseworthy	SA	34°32’53.8”S 138°47’08.3”E	380	404	loam over clay on rock
10		2020	Roseworthy	SA	same as 9	380	337	same as 9
11		2019	Roseworthy	SA	same as 9	380	246	same as 9
12		2017	Roseworthy	SA	same as 9	380	466	same as 9
13		2016	Roseworthy	SA	same as 9	380	602	same as 9
14		2015	Roseworthy	SA	same as 9	380	322	same as 9
15	BLYPRIM	2021	Blyth	SA	33°47’50.3”S 138°25’13.2”E	400	401	sandy loam
16		2020	Blyth	SA	same as 15	400	503	same as 15
17		2019	Blyth	SA	same as 15	400	189	same as 15
18		2018	Blyth	SA	same as 15	400	224	same as 15
19		2017	Blyth	SA	same as 15	400	331	same as 15
20		2016	Blyth	SA	same as 15	400	485	same as 15
21		2015	Blyth	SA	same as 15	400	353	same as 15
22	BIRCS4	2021	Birchip	VIC	35°45’54.0”S 142°43’35.8”E	304	256	sandy loam
23	LAMPRIM	2020	Lameroo	SA	35°20’52.4”S 140°36’18.9”E	314	356	non wetting sand over hard clay
24		2019	Lameroo	SA	same as 23	314	227	same as 23
25		2017	Lameroo	SA	same as 23	314	352	same as 23
26		2016	Lameroo	SA	same as 23	314	408	same as 23
27		2015	Lameroo	SA	same as 23	314	291	same as 23
28	KIG1X4	2019	Roseworthy	SA	34°32’53.8”S 138°47’08.3”E	380	246	loam over clay on rock
29		2018	Roseworthy	SA	same as 28	380	294	same as 28
30		2017	Roseworthy	SA	same as 28	380	466	same as 28
31		2016	Roseworthy	SA	same as 28	380	602	same as 28
32		2015	Roseworthy	SA	same as 28	380	322	same as 28
33	WOLPRIM	2018	Wolseley	SA	36°23’04.3”S 140°53’23.0”E	554	497	deep brown cracking clay
34		2017	Wolseley	SA	same as 33	554	607	same as 33
35		2015	Wolseley	SA	same as 33	554	331	same as 33

### 2.3 Genetic material evolvement and pedigree data

The NVBP commenced in 1993 with 23 introductions from gene banks in Europe and the Middle East. These were mostly landrace lines from existing collections with very little original passport data attached. There were also several older varieties, Blanchefleur and Languedoc, which were introduced into Australia as selections from European varieties in the 1960’s. This formed the basis of the breeding program and can be seen in the pedigrees of the genetic material as grandparents and great grandparents. This is particularly evident with introductions like IK 3 and IK 5 which initially provided rust (*Uromyces viciae fabae*) resistance genes to the breeding program and can been seen in the background of a large number of pedigrees. Crosses made from these lines appear commonly in the background of many lines still being used today. The genetic diversity within the program was greatly increased in the late 1990’s with collection trips to ICARDA in Syria and several eastern European collections, particularly the Vavilov Institute in Russia which had an extensive *Vicia* collection at this time. This broadened the genetic base of the breeding program offering greater diversity. The collection now includes over 1200 accessions from 38 countries. These accessions were all screened for individual traits and/or adaptation to Australian conditions and our Mediterranean environment in the Southern cropping zones. The traits assessed included disease resistance (Rust, *Ascochyta* and *Botrytis*), plant type, flower color, cotyledon color, hard seededness, adaptation to autumn/winter sowing, variations in flowering time and maturity, non-shattering pods, plant architecture and other traits that reflect domestication of plant species. This germplasm was then selected for desirable traits and progressed into the breeding program to introgress different combinations of these traits into elite breeding material producing the genetic material used in these experiments. The use of the pedigree has been an excellent approach to breakdown the historical relationships from the complex and extended families of this breeding material.

The pedigree file of the genetic material tested in the MET was assembled using the pedigree (Coster, 2022) and pedicure (Butler, 2019) R packages in the R statistical computing environment (R Core Team, 2022). The file contained four columns corresponding to the entry line, male parent, female parent, and level of selfing. The pedigree was checked to ensure that parents preceded their offspring, all parents appeared as entries, parents of base entries were set up to zero and, the pedigree did not contain duplicated, erroneous names and non-informative entries. The latter refers to lines which did not have phenotypic data and were not part of the set of ancestors. The final pedigree file contained 503 entries. Lines tested in the MET were traced back as many generations as information was available. This resulted in the following number of lines with the number of generations traced back provided in brackets: 41 (introductions, no parental information), 36 (1), 27 (2), 110 (3), 90 (4), 70 (5), 122 (6) and 7 (7). All lines underwent five generation of selfing, thus they were highly inbred with inbreeding coefficient >0.96. The degree of relatedness in the lines of the pedigree was not considered to be high with an average coefficient of coancestry of 0.13.

### 2.4 Statistical analyses

#### 2.4.1 Standard and pedigree multi-environment trial models

Yield data across trials was analyzed using linear mixed models that appropriately accounted for sources of variation derived from trials, variety by trial (environment) interactions, and spatial variation within each trial. The baseline model included a vector of fixed trial effects, vector of random genetic effects within trials, vector of random trial block effects to account for the structure of the experimental designs, and a vector of residual errors. Additional global or extraneous effects were added if required to account for the sources of spatial variation within each trial, and the correlation structures of errors were modelled according to autoregressive processes of order one in rows and columns (Gilmour et al., 1997). In this first stage, the pedigree information was not included, and this model is denoted by the standard MET model. The variance-covariance of the genetic by environment effects had a separable form where the between environment variance-covariance was first assumed to follow a diagonal (DIAG) model and lines were assumed to be independent between them. The DIAG model was then updated by a series of FA models of increasing order (Smith et al., 2001) to account for heterogeneity of covariances between pairs of environments.

Following Oakey et al. (2006, 2007), the (total) genetic effects were partitioned into additive and residual non-additive genetic effects in a pedigree MET model. The total genetic effects are used to indicate the commercial value of the lines, the additive effects are also known as breeding values and reveal the performance of the lines as parents, the residual genetic effects account for reduced or enhanced performance but are considered nonheritable. All the lines were the result of five generations of self-fertilization being considered highly homozygous, and consequently heterozygous dominance effects were assumed to be zero. Separable variance-covariance matrices were assumed for the additive and residual genetic by environment effects. The between environments variance-covariance matrices were firstly assumed to follow a DIAG model and then substituted by a series of FA models of increasing order for both the additive and residual genetic components. The inter-line relationships of the additive effects were accounted by the known additive relationship matrix. This matrix played a key role as the information of the pedigree was incorporated in the linear mixed models through its inverse. The ainverse function in ASReml-R software (Butler et al., 2017) uses the pedigree information to calculate the inverse of the additive relationship matrix (Meuwissen and Luo, 1992) with the adjustments of the inbreeding coefficient for the level of selfing. This inverse matrix was incorporated in the models through the vm function in ASReml-R software (Butler et al., 2017) in order to be used in the prediction of the additive genetic effects. The residual genetic effects were considered independent. A more detailed methodological description of the proposed statistical models can be found in **Supplementary Appendix 1**.

#### 2.4.2 Model selection and software

The number of loading factors was sequentially increased for standard and pedigree MET models and selected based on the lowest Akaike Information Criterion, AIC, (Akaike, 1974) and the percentage of genetic variance explained by the factors.

Residuals fulfilled the normality and homoscedasticity assumptions of the model. All models were fitted using the ASReml-R software (Butler et al., 2017) in the R statistical computing environment (R Core Team, 2022).

#### 2.4.3 Inference and predictions

The method of residual maximum likelihood (REML) was used for variance parameter estimation (Patterson and Thompson, 1971). Followed by the estimation of the variance parameters, the empirical best linear unbiased estimates (BLUEs) and empirical best linear predictors (BLUPs) were obtained for the fixed and random effects, respectively. Predictions of the total and additive genetic effects were evaluated within and across environments for specific and broad selections of commercial lines and parents. Predictions from the additive effects were summarized using the factor analytic selection tools (FAST) derived in Smith and Cullis (2018), which provided measures of overall performance (OP) and stability (RMSD) for each line. A more detailed description of the FAST methodology can be found in **Supplementary Appendix 1**.

## 3 Results

### 3.1 Model selection

A numerical summary of the standard and pedigree MET fitted models are presented in **Tables 2**, **3**. **Table 2** presents key information for the standard MET models. Model 1 was the baseline model which included a simple DIAG structure for the variance-covariance of the genetic effects between environments and independence between lines. In this first stage, the spatial variability at each trial was modelled and the spatial terms (**Supplementary Table S1**) were kept in the rest of the models (2-17). Models 2-7 included increasing order of FA models for the genetic component. **Table 3** displays the sequence of fitted pedigree MET models with the adjustment of the inbreeding coefficient for five generations of selfing for each line. Model 8 included a DIAG structure for the variance-covariance of the additive and non-additive genetic effects between environments. The trials 2017LAMPRIM, 2018BLYPRIM, 2021VetchRank2, and 2021WMG were dropped from the non-additive component as their corresponding variances were estimated to be zero. The remaining models (9-17) included increasing order of FA models for the additive and non-additive genetic components.

**Table 2:** Summary of the models fitted in the standard multi-environment trial analysis including environment genetic variance structure (*G_e_*), total number of variance parameters (v), loglikelihood value (LL), Akaike Information Criterion (AIC), and percentage of genetic variance accounted for by the factor analytic regression (%*G_e_* FA).

Model	*G_e_*	v	LL	AIC	% *G_e_* FA
1	DIAG^a^	161	8186.92	-16051.83	–
2	FA1^b^	196	8654.90	-16917.81	61.59
3	FA2	228	8820.25	-17184.49	76.72
4	FA3	257	8889.01	-17264.03	83.11
5	FA4	287	8918.29	-17262.58	87.73
6	FA5	312	8955.20	-17286.40	93.44
7	FA6	342	8984.80	-17285.61	95.14

a DIAG denotes a diagonal matrix structure.

b FAk denotes a factor analytic structure of k factors.

**Table 3:** Summary of the models fitted in the pedigree multi-environment analysis including environment genetic variance structure for the additive (G_a_) and non-additive (G_p_) component, number of variance parameters (v), loglikelihood value (LL), Akaike Information Criterion (AIC), and percentage of genetic additive and non-additive variance accounted for by the factor analytic regression (% G_a_FA, and % G_p_FA, respectively).

Model	G_a_	G_p_	v	LL	AIC	% G_a_FA	% G_p_FA
8	DIAG^b^	DIAG	192	8351.74	-16319.47	–	–
9	FA1^c^	DIAG	224	8799.79	-17151.59	67.06	–
10	FA1	FA1	250	8918.34	-17336.67	71.96	64.95
11	FA2	FA1	267	8993.18	-17452.35	78.16	85.70
12	FA3	FA1	296	9049.62	-17507.25	86.36	81.57
13	FA4	FA1	326	9085.72	-17519.45	90.40	83.62
14	FA3	FA2	327	9085.46	-17516.91	90.02	84.40
15	FA3	FA3	350	9109.94	-17519.88	91.15	96.17
16	FA4	FA2	350	9123.25	-17546.5	94.62	95.21
17^a^	FA4	FA3	376	9145.80	-17539.60	95.44	96.33

a Final model selected.

b DIAG denotes a diagonal matrix structure.

c FAk denotes a factor analytic structure of k factors.

On comparing the AIC values between **Tables 2**, **3**, it is clear that including the pedigree information improved the fit of the models. From **Table 2**, Model 6 obtained the lowest AIC, but it was inferior to Models 16 and 17 which were the best fitted models. Model 17 was finally chosen to produce predictions. This model explained over 95% of the additive and non-additive genetic variance. Furthermore, care should be taken when fitting an FA model with only one or two factors (non-additive component in Model 16) as scenarios where there are zero specific variances for pairs of environments could lead to perfect or very high genetic correlations between those environments.

### 3.2 Genetic variance-covariance estimation and clustering of environments

The additive and non-additive genetic variance-covariance matrices were estimated based on Model 17. The proportion of the additive genetic variance in relation to the total genetic variance at each environment is presented in **Supplementary Table S2**. The additive variance explained over 70% of the total genetic variance for all the environments except in seven of them. This means that for the majority of the environments, most of the genetic variance was due to the heritable component of the trait. The high proportion of the total genetic variance explained by the additive genetic variance led to a high correlation between the total and the additive genetic effects.

**Figure 2:**
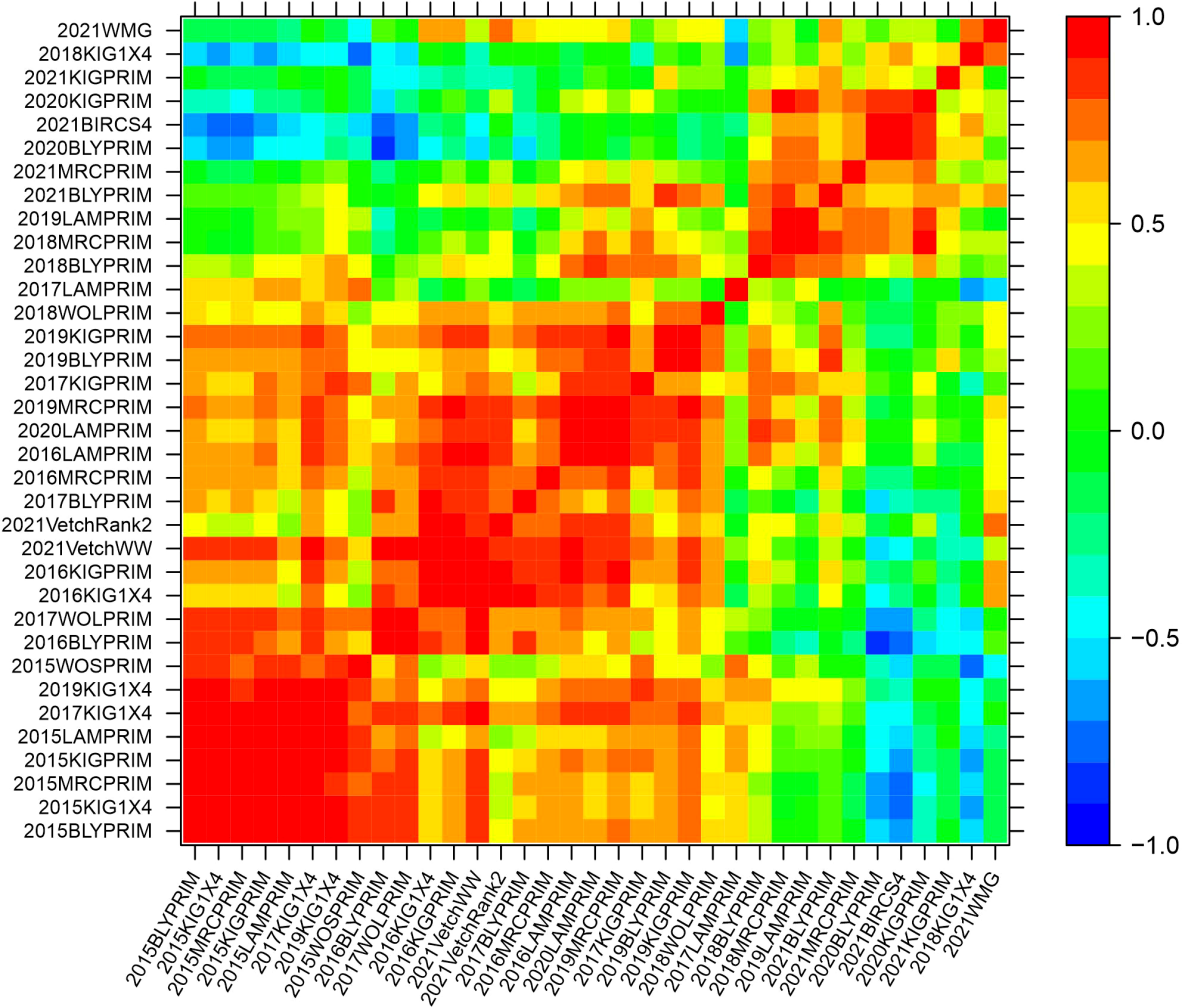
Heat map representation of the additive genetic correlation matrix. The color scale indicates the degree of correlation which goes from -1 (dark blue) to 1 (red).

**Figure 3:**
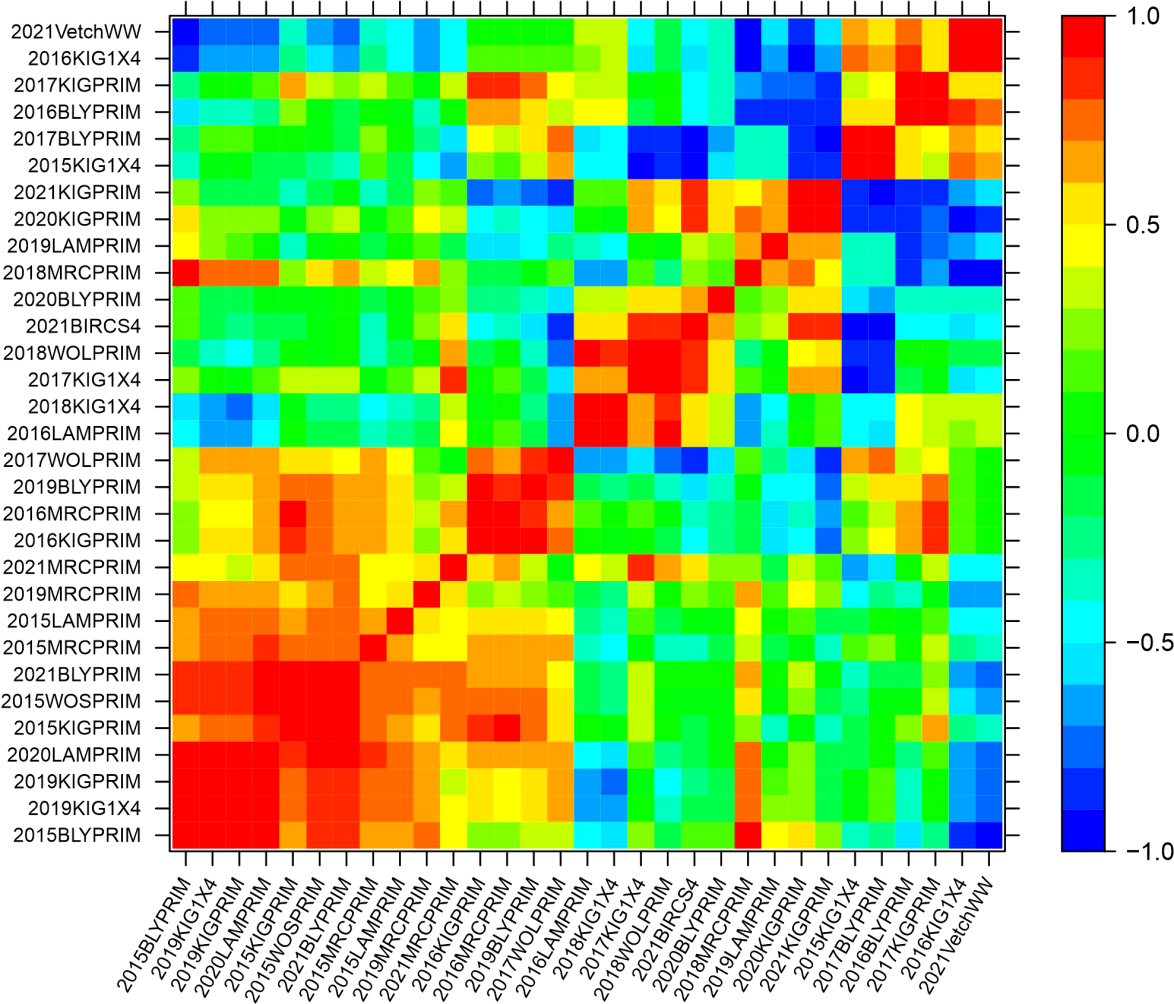
Heat map representation of the non-additive genetic correlation matrix. The color scale indicates the degree of correlation which goes from -1 (dark blue) to 1 (red).

The genetic variance-covariance matrices were converted into genetic correlation matrices to inspect the rankings of the genetic effects. The genetic correlations indicate the degree of agreement in the rankings of varieties between pairs of environments. High and positive correlations (over 0.7) refer to a good agreement in the rankings, while high negative correlations indicate reverse order. Agglomerative hierarchical clustering was performed on the additive and non-additive genetic correlation matrices to classify environments into clusters. Groups of environments were defined to ensure that the average pairwise genetic correlation between groups did not exceed 0.5. Five clusters were classified based on the additive genetic correlation matrix with 23, 8, 2, 1 and 1 elements (**Supplementary Figure 1**). In this classification, three clusters contained only one or two environments implying that they were mainly classified in two major clusters. Based on the non-additive genetic correlation matrix, a classification of five clusters was obtained with 15, 6, 5, 4 and 1 environments (**Supplementary Figure 2**). Heat map representations of the additive and non-additive genetic correlation matrices are presented in **Figures 2**, **3**, respectively. The order of the environments in the axes is presented according to the agglomerative hierarchical clustering results. The non-additive genetic correlations between environments presented in **Figure 3** were lower when compared to **Figure 2**. The range of the additive correlations went from -0.83 to 0.99 with 80% of the correlations being positive and 27% being greater than 0.7. The range of the non-additive correlations went from -0.99 to 0.99 with 58% of the correlations being positive and 15% being greater than 0.7.

### 3.3 Selection of lines

The total genetic effects were predicted using Model 6, based on a minimum AIC for the models without pedigree, and Model 17, the final model selected. The objective was to compare the total genetic effects for the analyses with and without pedigree information. A high correlation (ranging from 0.71-0.93) was found for the total genetic effects under both models for all the environments (**Supplementary Figure 3**). Results (described below) were based on predictions from Model 17 as this model provided lower AIC and enabled us to select lines for their commercial value and as progenitors for future crosses.

The total genetic effects were inspected within and across environments for ranking varieties according to their commercial value. The line 38222 had the highest average total effect with positive individual total effects for all the environments indicating an extremely good broad adaptation across environments. Another interesting line was 37107 which had the second highest average total effect and performed extremely well in environments with acid soils like 2021WW, 2021Rank2 and 2021WMG. However, in years with extremely low rainfall like 2015, its adaptation was reduced showing a notable loss of production. The lines 38578, 37695, 38562, 37625 and 37626 had a high commercial value across environments with positive total effects across all the trials where they were sown, indicating a broad adaptation across environments.

The additive effects of the lines which were not sown in the trials but were present in the pedigree were also investigated. These effects are predictable due to the relationships established between the lines in the pedigree and they are used for the selection of varieties as parents. When inspecting the average additive genetic effects, the line 37107 had the highest average additive BLUPs and it was the best parent in 22% of the trials. The variety 38222 was one of the best parents as its average additive genetic effect was the second largest among all the lines and its performance was always positive across all trials. Therefore, 38222 was a parent with good broad adaptation and 37107 showed an extremely well adaptation to some specific environments. The lines 38562 and 37482 also had a high average additive genetic effect with positive individual additive effects for all the environments indicating a broad adaptation. The highest single individual additive effect was found for the introduction VAR 2 in 2016KIG1x4. This line was also a parent in two other lines in the top 20 individuals, making it a line of interest for the crossing program. This line may only display specific adaptation to limited environments, but it has the potential to offer high yield potential as a parent in others. The potential of the lines with exceptional yields in good conditions but low adaptation to poor conditions could be exploited to breed vetch lines for higher rainfall environments which are not currently targeted by the NVBP.

To further investigate the additive genetic effects, we used the OP and RMSD measures. These tools could be used as the first factor accounted for 50% of the additive genetic variance with most of the loadings being positive except for only 5 out of 35 which had small negative values. The top 20 parents based on the highest OP values are shown in **Table 4**. We compared the OP values with the average additive effects obtaining a correlation of 0.97. Furthermore, among the parents selected in **Table 4**, we found 16 lines in common when they were ranked based the highest average additive effect. The RMSD was used to distinguish which of these parents were the most stable across environments indicating a good broad adaptation. For instance, the lines 38397, 38221, 38563, 37654, and 38578 were among the top ten parents with high stability. When comparing 38222 and 37107, we found that 38222 was more stable congruently with the inspection of their individual BLUPs. Varieties were ranked according to their RMSD. In terms of stability, the lines 38397 and 37623 were among the top 20 parents for both their stability and OP. The regression plots associated to the first loading were inspected to visualize the OP and the RMSD values to aid with interpretation of the results. As an example, **Supplementary Figure 4** displays these regressions for the varieties in **Table 4**.

**Table 4:** Summary of 20 best lines as parents for future crosses ranked on their overall performance (OP), also displaying their stability measure (RMSD) and average additive effects ( ${\bar{\tilde{a}}}_{î}$ ).

Line	OP	RMSD	${\bar{\tilde{a}}}_{î}$	Line	OP	RMSD	${\bar{\tilde{a}}}_{î}$
38397	0.236	0.030	0.224	38684-2	0.185	0.059	0.201
37665	0.224	0.118	0.242	37877	0.183	0.051	0.198
38221	0.213	0.079	0.226	37907	0.181	0.111	0.215
37107	0.211	0.213	0.259	38535	0.178	0.140	0.120
37666	0.211	0.142	0.228	37623	0.175	0.029	0.154
38563	0.208	0.081	0.234	34869	0.173	0.089	0.159
38222	0.206	0.145	0.246	38530	0.172	0.120	0.186
37654	0.191	0.080	0.210	37626	0.164	0.129	0.189
38578	0.189	0.091	0.221	38727	0.160	0.080	0.178
38537	0.185	0.121	0.124	38562	0.159	0.060	0.189

In summary, the use of the pedigree has allowed to breakdown the relationship between the complex and extended families of this breeding material. The results of this study have improved the ability of breeders to select superior commercial lines and parents for future crosses for broad and specific adaptation. Furthermore, the OP and RMSD measures further exploits these results to identify the most stable parents across environments

## 4 Discussion

In this study, the pedigree MET models have been used for the first time to analyze yield in a breeding program for common vetch. These models were successfully implemented with the inclusion of the pedigree information substantially improving the fit to the data and the additive component explaining a big proportion of the total genetic variance in most of the trials. The additive and total genetic effects were inspected within and across environments and the FAST approach of Smith and Cullis (2018) was used to summarize and interpret the additive genetic effects.

The typical statistical methodology used for yield analysis in common vetch breeding programs is still mostly based in early methods such as analysis of variance or simple variance components mixed models (Berger et al., 2002; Georgieva et al., 2015; Aydemir et al., 2019; Dong et al., 2019). These models assume variance structures which may not be realistic and do not allow to explore patterns in the G x E interaction (Kempton, 1984; Smith et al., 2005). To overcome this limitation, additive main effects and multiplicative interaction models (AMMI) have been used to investigate these patterns through the decomposition of the G x E interaction in principal components (Greveniotis et al., 2021). However, they require balanced data and assume fixed varieties instead of random, being the latter the recommended approach when the aim of the analysis is selection (Smith et al., 2005). None of these typical statistical techniques have the ability to account for the relationships between lines derived from their inheritance of common ancestors.

The pedigree MET models used in this study are much more flexible in comparison with the current methodology described above and present several advantages. They allow to handle unbalanced data, typical in MET studies since the varieties with the lowest performance are substituted by new ones over the years. They enable to fit a realistic genetic variance structure accounting for interline relationships, heterogeneity of genetic variance and covariance between environments, and simultaneously model the spatial variation and heterogeneity of residuals at each trial. In addition, these models can distinguish lines for their commercial value or/and as parents for future crosses. Furthermore, the exploration of the FA (loadings and scores) enables the investigation of G x E patterns in the additive and non-additive component. Due to these benefits, the FA approach of Smith et al. (2001) is the statistical method adopted in all major plant breeding programs in Australia where pedigree information is available (Tolhurst et al., 2019).

In this study, the pedigree MET models presented lower AIC values and thus a better fit to the data compared to the standard MET models. The superiority of the fit of the pedigree MET models was also obtained in other crop breeding programs such as those for yield production in barley (Kelly et al., 2009), oil and grain yield production in canola (Beeck et al., 2010), or resistance to powdery scab in potato (Paget et al., 2014). Furthermore, simulation studies in Oakey (2008) demonstrated that incorporating the pedigree information resulted in total and additive genetic effects with lower mean square error of predictions. Kelly et al. (2009) also suggested that the estimates of line performance are more accurate when including the correlated information from relatives.

A major benefit of the inclusion of the pedigree is the ability to select varieties for their commercial release or/and potential as parents in future crosses. In this study, the additive and total genetic effects were inspected within and across environments. Furthermore, the OP and stability measures derived in Smith and Cullis (2018) were used to summarize and interpret the potential of the varieties as parents. Results in our study provided growers with a better understanding in the selection of lines for future crossing. For instance, we were able to determine the top progenitors (**Table 4**) and among them to distinguish the most stable ones, e.g., 38397, 38221, 38563, and 37654. The specific adaptation of progenitors to the environments were investigated using their individual additive BLUPs. For example, 37107 performed extremely well in environments with acid soils and good rainfall or line VAR2 in the 2016KIG1x4 environment. Other breeding studies for other crops (Tolhurst et al., 2019; Martins Oliveira et al., 2020; Sjoberg et al., 2021) have reported the benefits to use the OP and RMSD measures of Smith and Cullis (2018) to identify superior varieties. To the best of the authors’ knowledge, our study is the first common vetch breeding MET which has implemented the FAST tools for selection of superior and stable parents.

In conclusion, in the absence of genetic marker information, the use of the pedigree to model the relationship between lines represents a plausible and cost-efficient alternative as it has been demonstrated in this study. The inclusion of the pedigree has been successfully implemented for the first time to analyze yield in common vetch and provided vetch breeders with improved ability to select progenitors and lines for commercial release.

## Data availability statement

The raw data supporting the conclusions of this article will be made available by the authors, without undue reservation.

## Author contributions

SN designed and implemented the experiments. SN collected all data from the trials. IM curated and analyzed the data. IM wrote the first draft of the article and led the writing process. JT and SN significantly contributed to the conceptualization of the study, manuscript reviewing and editing. All authors contributed to the article and approved the submitted version.

## Footnotes

1
www.fao.org/faostat

## References

[B1] Aguilar I. Misztal I. Johnson D. L. Legarra A. Tsuruta S. Lawlor T. J. (2010). Hot topic: A unified approach to utilize phenotypic, full pedigree, and genomic information for genetic evaluation of Holstein final score. J. dairy Sci. 93, 743–752. doi: 10.3168/jds.2009-2730 20105546

[B2] Akaike H. (1974). A new look at the statistical model identification. IEEE Trans. automatic control 19, 716–723. doi: 10.1109/TAC.1974.1100705

[B3] Asfaw A. Aderonmu D. S. Darkwa K. De Koeyer D. Agre P. Abe A.. (2021). Genetic parameters, prediction, and selection in a white Guinea yam early-generation breeding population using pedigree information. Crop Sci. 61, 1038–1051. doi: 10.1002/csc2.20382 33883753 PMC8048640

[B4] Aydemir S. K. Karakoy T. Kokten K. Nadeem M. A. (2019). Evaluation of yield and yield components of common vetch (Vicia sativa L.) genotypes grown in different locations of Turkey by GGE biplot analysis. Appl. Ecol. Environ. Res. 17, 15203–15217. doi: 10.15666/aeer/1706_1520315217

[B5] Beeck C. P. Cowling W. A. Smith A. B. Cullis B. R. (2010). Analysis of yield and oil from a series of canola breeding trials. Part I. Fitting factor analytic mixed models with pedigree information. Genome 53, 992–1001. doi: 10.1139/G10-051 21076515

[B6] Berger J. D. Robertson L. D. Cocks P. S. (2002). Genotype × environment interaction for yield and other plant attributes among undomesticated Mediterranean Vicia species. Euphytica 126, 421–435. doi: 10.1023/A:1019938300971

[B7] Bergsma R. Kanis E. Knol E. F. Bijma P. (2008). The contribution of social effects to heritable variation in finishing traits of domestic pigs (Sus scrofa). Genetics 178, 1559–1570. doi: 10.1534/genetics.107.084236 18245326 PMC2391867

[B8] Brown D. Tier B. Reverter A. Banks R. Graser H. (2000). OVIS: A multiple trait breeding value estimation program for genetic evaluation of sheep. Wool Technol. Sheep Breed. 48, 285–297.

[B9] Butler D. (2019). Pedicure: pedigree tools. R Package version 2.0.0.

[B10] Butler D. Cullis B. Gilmour A. Gogel B. Thompson R. (2017). ASReml-R reference manual version 4 (UK: VSN International Ltd, Hemel Hempstead, HP1 1ES).

[B11] Coster A. (2022). pedigree: pedigree functions. R Package version 1.4.2.

[B12] Cowling W. A. Castro-Urrea F. A. Stefanova K. T. Li L. Banks R. G. Saradadevi R.. (2023). Optimal contribution selection improves the rate of genetic gain in grain yield and yield stability in spring canola in Australia and Canada. Plants 12, 383. doi: 10.3390/plants12020383 36679096 PMC9863350

[B13] Cullis B. R. Jefferson P. Thompson R. Smith A. B. (2014). Factor analytic and reduced animal models for the investigation of additive genotype-by-environment interaction in outcrossing plant species with application to a Pinus radiata breeding programme. Theor. Appl. Genet. 127, 2193–2210. doi: 10.1007/s00122-014-2373-0 25145447

[B14] Dalias P. Neocleous D. (2017). Comparative analysis of the nitrogen effect of common agricultural practices and rotation systems in a rainfed mediterranean environment. Plants 6, 61. doi: 10.3390/plants6040061 29211012 PMC5750637

[B15] Daly J. Anderson K. Ankeny R. Harch B. Hastings A. Rolfe J.. (2015). Australia's agricultural future. Aust. Council Learned Academies (ACOLA).

[B16] Dong R. Shen S. H. Jahufer M. Z. Z. Dong D. K. Luo D. Zhou Q.. (2019). Effect of genotype and environment on agronomical characters of common vetch (Vicia sativa L.). Genet. Resour. Crop Evol. 66, 1587–1599. doi: 10.1007/s10722-019-00789-3

[B17] Durel C. E. Laurens F. Fouillet A. Lespinasse Y. (1998). Utilization of pedigree information to estimate genetic parameters from large unbalanced data sets in apple. Theor. Appl. Genet. 96, 1077–1085. doi: 10.1007/s001220050842

[B18] Dutkowski G. W. Silva J. C. E. Gilmour A. R. Lopez G. A. (2002). Spatial analysis methods for forest genetic trials. Can. J. For. Res. 32, 2201–2214. doi: 10.1139/x02-111

[B19] Fanning J. Linsell K. Mckay A. Gogel B. Munoz Santa I. Davey R.. (2018). Resistance to the root lesion nematodes Pratylenchus thornei and P. neglectus in cereals: Improved assessments in the field. Appl. Soil Ecol. 132, 146–154. doi: 10.1016/j.apsoil.2018.08.023

[B20] Gao L. Bryan B. A. (2017). Finding pathways to national-scale land-sector sustainability. Nature 544, 217–222. doi: 10.1038/nature21694 28406202

[B21] Georgieva N. Nikolova I. Kosev V. (2015). Stability analysis for seed yield in vetch cultivars. Emirates J. Food Agric. 27, 903–910. doi: 10.9755/ejfa.2015-04-172

[B22] Gilmour A. Cullis B. Verbyla A. (1997). Accounting for natural and extraneous variation in the analysis of field experiments. J. Agricultural Biological Environ. Stat 2, 269–293. doi: 10.2307/1400446

[B23] Greveniotis V. Bouloumpasi E. Zotis S. Korkovelos A. Ipsilandis C. G. (2021). Assessment of interactions between yield components of common vetch cultivars in both conventional and low-input cultivation systems. Agriculture 11, 369. doi: 10.3390/agriculture11040369

[B24] Henchion M. Hayes M. Mullen A. M. Fenelon M. Tiwari B. (2017). Future protein supply and demand: strategies and factors influencing a sustainable equilibrium. Foods 6, 53. doi: 10.3390/foods6070053 28726744 PMC5532560

[B25] Henderson C. R. (1976). A simple method for computing the inverse of a numerator relationship matrix used in prediction of breeding values. Biometrics 32, 69–83. doi: 10.2307/2529339

[B26] Huang Y. F. Gao X. L. Nan Z. B. Zhang Z. X. (2017). Potential value of the common vetch (Vicia sativa L.) as an animal feedstuff: a review. J. Anim. Physiol. Anim. Nutr. 101, 807–823. doi: 10.1111/jpn.12617 28066943

[B27] Kaseja K. Mucha S. Smith E. M. Yates J. Banos G. Conington J. (2023). Including genotypic information in genetic evaluations increases the accuracy of sheep breeding values. J. Anim. Breed. Genet. 00, 1–10. doi: 10.1111/jbg.12771 PMC10952277 37002932

[B28] Kelly A. M. Cullis B. R. Gilmour A. R. Eccleston J. A. Thompson R. (2009). Estimation in a multiplicative mixed model involving a genetic relationship matrix. Genet. Selection Evol. 41, 1–9. doi: 10.1186/1297-9686-41-33 PMC2686677 19356255

[B29] Kempton R. A. (1984). The use of biplots in interpreting variety by environment interactions. J. Agric. Sci. 103, 123–135. doi: 10.1017/S0021859600043392

[B30] Lal R. (2016). Feeding 11 billion on 0.5 billion hectare of area under cereal crops. Food Energy Secur. 5, 239–251. doi: 10.1002/fes3.99

[B31] Mariotti M. Masoni A. Ercoli L. Arduini I. (2006). Forage potential of winter cereal/legume intercrops in organic farming. Ital. J. Agron. 1, 403–412. doi: 10.4081/ija.2006.403

[B32] Martins Oliveira I. C. Soler Guilhen J. H. De Oliveira Ribeiro P. C. Gezan S. A. Schaffert R. E. Ferreira Simeone M. L.. (2020). Genotype-by-environment interaction and yield stability analysis of biomass sorghum hybrids using factor analytic models and environmental covariates. Field Crops Res. 257, 107929. doi: 10.1016/j.fcr.2020.107929

[B33] Mathews K. L. Chapman S. C. Trethowan R. Pfeiffer W. Van Ginkel M. Crossa J.. (2007). Global adaptation patterns of Australian and CIMMYT spring bread wheat. Theor. Appl. Genet. 115, 819–835. doi: 10.1007/s00122-007-0611-4 17768603

[B34] Matić R. Nagel S. Kirby G. Young I. Smith K. (2007). Vetch breeding and vetch use in Australia. Zbornik radova Instituta za ratarstvo i povrtarstvo 44, 55–63.

[B35] Matić R. Nagel S. Robertson S. Young I. Mihailović V. Mikić A.. (2005). Vetch (Vicia spp) expansion and use in Australia. Biotechnol. Anim. Husbandry 21, 203–207. doi: 10.2298/BAH0502203M

[B36] Maxted N. Bennett S. J. (2001). Plant genetic resources of legumes in the Mediterranean (Netherlands: Springer Science & Business Media).

[B37] Melnikova E. Kabanov A. Nikitin S. Somova M. Kharitonov S. Otradnov P.. (2021). Application of genomic data for reliability improvement of pig breeding value estimates. Animals 11, 1557. doi: 10.3390/ani11061557 34071766 PMC8229591

[B38] Meuwissen T. H. E. Luo Z. (1992). Computing inbreeding coefficients in large populations. Genet. Selection Evol. 24, 305–313. doi: 10.1051/gse:19920402

[B39] Mihailovich V. Mikic A. Pataki I. Katic S. Karaggic D. Milosevic M. (2005). Yield and forage yield components in winter vetch cultivars. Acta Agriculturae Serbia IX, 407–411.

[B40] Oakey H. Verbyla A Pitchford W. Cullis B. Kuchel H. (2006). Joint modeling of additive and non-additive genetic line effects in single field trials. Theoretical and Applied Genetics 113, 809–819. doi: 10.1007/s00122-006-0333-z 16896718

[B41] Oakey H. Verbyla A. P. Cullis B. R. Wei X. Pitchford W. S. (2007). Joint modeling of additive and non-additive (genetic line) effects in multi-environment trials. Theoretical and Applied Genetics 114, 1319–1332. doi: 10.1007/s00122-007-0515-3 17426958

[B42] Oakey H. (2008). Incorporating pedigree information into the analysis of agricultural genetic trials (Australia:University of Adelaide).

[B43] Paget M. F. Alspach P. A. Genet R. A. Apiolaza L. A. (2014). Genetic variance models for the evaluation of resistance to powdery scab (Spongospora subterranea f. sp. subterranea) from long-term potato breeding trials. Euphytica 197, 369–385. doi: 10.1007/s10681-014-1073-9

[B44] Pala M. Armstrong E. Johansen C. (2000). “The role of legumes in sustainable cereal production in rainfed areas,” in Linking Research and Marketing Opportunities for Pulses in the 21st Century. (Netherlands: Springer).

[B45] Panter D. M. Allen F. L. (1995). Using best linear unbiased predictions to enhance breeding for yield in soybean: I. Choosing parents. Crop Sci. 35, 397–405. doi: 10.2135/cropsci1995.0011183X003500020020x

[B46] Patterson H. D. Silvey V. Talbot M. Weatherup S. T. C. (1977). Variability of yields of cereal varieties in UK trials. J. Agric. Sci. 89, 239–245. doi: 10.1017/S002185960002743X

[B47] Patterson H. Thompson R. (1971). Recovery of inter-block information when block sizes are unequal. Biometrika 58, 545–554. doi: 10.1093/biomet/58.3.545

[B48] Potokina E. Blattner F. Alexandrova T. Bachmann K. (2002). AFLP diversity in the common vetch (Vicia sativa L.) on the world scale. Theor. Appl. Genet. 105, 58–67. doi: 10.1007/s00122-002-0866-8 12582562

[B49] Quaas R. L. Pollak E. J. (1980). Mixed model methodology for farm and ranch beef cattle testing programs. J. Anim. Sci. 51, 1277–1287. doi: 10.2527/jas1981.5161277x

[B50] Real D. Oldham C. M. Nelson M. N. Croser J. Castello M. Verbyla A.. (2014). Evaluation and breeding of tedera for Mediterranean climates in southern Australia. Crop Pasture Sci. 65, 1114–1131. doi: 10.1071/CP13313

[B51] Sissons M. Kadkol G. Taylor J. (2020). Genotype by environment effects on durum wheat quality and yield-implications for breeding. Crop Breeding Genet. Genomics 2, e200018. doi: 10.20900/cbgg20200018

[B52] Sjoberg S. M. Carter A. H. Steber C. M. Garland Campbell K. A. (2021). Application of the factor analytic model to assess wheat falling number performance and stability in multienvironment trials. Crop Sci. 61, 372–382. doi: 10.1002/csc2.20293

[B53] Smith A. B. Cullis B. R. (2018). Plant breeding selection tools built on factor analytic mixed models for multi-environment trial data. Euphytica 214, 143–161. doi: 10.1007/s10681-018-2220-5

[B54] Smith A. Cullis B. Thompson R. (2001). Analyzing variety by environment data using multiplicative mixed models and adjustments for spatial field trend. Biometrics 57, 1138–1147. doi: 10.1111/j.0006-341X.2001.01138.x 11764254

[B55] Smith A. Cullis B. R. Thompson R. (2005). The analysis of crop cultivar breeding and evaluation trials: an overview of current mixed model approaches. J. Agric. Sci. 143, 449–462. doi: 10.1017/S0021859605005587

[B56] R Core Team (2022). R: A Language and Environment for Statistical Computing (Vienna, Austria: R Foundation for Statistical Computing).

[B57] Tolhurst D. J. Mathews K. L. Smith A. B. Cullis B. R. (2019). Genomic selection in multi-environment plant breeding trials using a factor analytic linear mixed model. J. Anim. Breed. Genet. 136, 279–300. doi: 10.1111/jbg.12404 31247682

[B58] Unkovich M. J. Pate J. S. Sanford P. (1997). Nitrogen fixation by annual legumes in Australian Mediterranean agriculture. Aust. J. Agric. Res. 48, 267–293. doi: 10.1071/A96099

[B59] Vasilakoglou I. Dhima K. Lithourgidis A. Eleftherohorinos I. (2008). Competitive ability of winter cereal–common vetch intercrops against sterile oat. Exp. Agric. 44, 509–520. doi: 10.1017/S0014479708006728

[B60] Wang Q. Zhang C. Li J. Wu X. Long Y. Su Y. (2021). Intercropping vicia sativa L. Improves the moisture, microbial community, enzyme activity and nutrient in rhizosphere soils of young kiwifruit plants and enhances plant growth. Horticulturae 7, 335. doi: 10.3390/horticulturae7100335

[B61] Welham S. J. Gogel B. J. Smith A. B. Thompson R. Cullis B. R. (2010). A comparison of analysis methods for late-stage variety evaluation trials. Aust. New Z. J. Stat 52, 125–149. doi: 10.1111/j.1467-842X.2010.00570.x

[B62] Zhang M. Luo H. Xu L. Shi Y. Zhou J. Wang D.. (2022). Genomic selection for milk production traits in xinjiang brown cattle. Animals 12, 136. doi: 10.3390/ani12020136 35049759 PMC8772551

[B63] Zhang J. Peng S. Andrews M. Liu C. Shang Y. Li S.. (2021). Rhizobium changzhiense sp. nov., isolated from effective nodules of Vicia sativa L. @ in North China. Int. J. Systematic Evolutionary Microbiol. 71, 4724. doi: 10.1099/ijsem.0.004724 33661090

