# Supplementary material for: Incorporating the pedigree information in multi-environment trial analyses for improving common vetch
Source: Front Plant Sci. 2023 Aug 16;14:1166133. doi: 10.3389/fpls.2023.1166133 (PMC10467272; doi:10.3389/fpls.2023.1166133)
Supplement: Supplementary file 1 [file DataSheet_1.zip › Data Sheet 1/Appendix I.docx]

**Appendix I: Extended explanation of the statistical methodology**

**Standard multi-environment trial models**

The vector$\boldsymbol{y}$of the $n$ observed yield responses across the $t$ trials (environments) was analysed using the following linear mixed model:

| $\boldsymbol{y}=\boldsymbol{X\tau}+\boldsymbol{Z}_{g}\boldsymbol{g}+\boldsymbol{Z}_{u}\boldsymbol{u}+\boldsymbol{e}$ | (1) |
| --- | --- |

where $\boldsymbol{\tau}$=$\left( \boldsymbol{\tau}_{1}^{T}, \boldsymbol{\tau}_{2}^{T},\ldots\boldsymbol{\tau}_{t}^{T} \right)^{T}$is the vector of fixed effects containing the trial means and terms to capture global trends at each trial, $\boldsymbol{X}$is its associated design matrix, $\boldsymbol{g}$ = $\left( \boldsymbol{g}_{1}^{T}, \boldsymbol{g}_{2}^{T}, \ldots, \boldsymbol{g}_{t}^{T} \right)^{\mathbf{T}}$is the vector of random genetic effects of the $m$ lines present in the $t$ trials, $\boldsymbol{Z}_{g}$is its associated design matrix, ${\boldsymbol{u}= \left( \boldsymbol{u}_{1}^{T}, \boldsymbol{u}_{2}^{T}, \ldots, \boldsymbol{u}_{r}^{T} \right)}^{T}$ is the vector of random non-genetic effects consisting in block effects for each trial and additional model terms to capture extraneous specific trial variation, with associated design matrix$\boldsymbol{Z}_{u}$, and ${\boldsymbol{e}= \left( \boldsymbol{e}_{1}^{T}, \boldsymbol{e}_{2}^{T}, \ldots, \boldsymbol{e}_{t}^{T} \right)}^{T}$ is the vector of residual errors partitioned by trials.

Initially, a separable variance-covariance of the form $\mathrm{var}\left( \boldsymbol{g} \right)=\boldsymbol{G}_{e}\otimes\boldsymbol{I}_{m}$ was assumed where the variance-covariance of the genetic effects between environments ($\boldsymbol{G}_{e})$was a diagonal (DIAG) matrix which accounted for different variances between environments and no genetic correlation between pairs of environments and the $m$ lines were independent. Ideally, an unstructured form of $\boldsymbol{G}_{e}$ is desired, which enables modelling the heterogeneity of genetic variances between environments and heterogeneity of genetic covariances between pairs of environments. Due to the large number of trials, a parsimonious approximation of the unstructured $\boldsymbol{G}_{e}$ was sought using the factor analytic (FA) approach of Smith et al. (2001). With this approach $\boldsymbol{G}_{e}$ becomes:

| $\boldsymbol{G}_{e}=\boldsymbol{\Lambda}_{e}\boldsymbol{\Lambda}_{e}^{T}+\boldsymbol{\Psi}_{e}$ | (2) |
| --- | --- |

where $\boldsymbol{\Lambda}_{e}=\left[ \boldsymbol{\lambda}_{e_{1}}, \boldsymbol{\lambda}_{e_{1}}, \ldots\boldsymbol{\lambda}_{e_{k}} \right]$ is the matrix of loadings and $\boldsymbol{\Psi}_{e}$is the diagonal matrix of specific trial variances.

The joint distribution of the random effects $\left( \boldsymbol{g}, \boldsymbol{u}, \boldsymbol{e} \right)$ was assumed to be Gaussian with zero mean and pairwise independent components. The $\mathrm{var}\left( \boldsymbol{u} \right)={\oplus_{i=1}^{r}\sigma_{u_{i}}^{2}\boldsymbol{I}}_{n_{u_{i}}}$and $\mathrm{var}\left( \boldsymbol{e} \right)=$ $\oplus_{i=1}^{t}\sigma_{i}^{2}\boldsymbol{\Sigma}\left( \rho_{ci} \right)\otimes\boldsymbol{\Sigma}\left( \rho_{ri} \right)$, where $\sigma_{u_{i}}^{2}$ denotes the variance associated to $\boldsymbol{u}_{i}$, $\boldsymbol{I}$ denotes the identity matrix,$\sigma_{i}^{2}$is the trial error variance and $\boldsymbol{\Sigma}\left( \rho_{ri} \right)\otimes\boldsymbol{\Sigma}\left( \rho_{ci} \right)$ refers to an autoregressive process of order one in the column and row directions in the $i^{th}$ trial.

**Pedigree multi-environment trial models**

Following Oakey et al (2006, 2007), the $\boldsymbol{g}$ (total) genetic effects of the lines present in the pedigree were partitioned into the additive genetic effect, $\boldsymbol{a}$, and residual non-additive genetic effects, $\boldsymbol{p}$. The linear mixed model to analyze the data including the pedigree information was:

| $\boldsymbol{y}=\boldsymbol{X\tau}+\boldsymbol{Z}_{g}(\boldsymbol{a}+\boldsymbol{p})+\boldsymbol{Z}_{u}\boldsymbol{u}+\boldsymbol{e}$ | (3) |
| --- | --- |

where all model terms are defined as in Eqn. 1 and $\boldsymbol{g}$ is replaced by $\boldsymbol{a}+\boldsymbol{p}$.

Separable variance-covariance matrices of the form $\mathrm{var}\left( \boldsymbol{a} \right)=\boldsymbol{G}_{a}\otimes\boldsymbol{A}$ and $\mathrm{var}\left( \boldsymbol{p} \right)=\boldsymbol{G}_{p}\otimes\boldsymbol{I}_{m}$ were assumed, where the $\boldsymbol{G}_{a}$ and $\boldsymbol{G}_{p}$are between trial unstructured variance-covariance matrices for the additive and non-additive genetic effects, respectively. $\boldsymbol{A}$ is the known additive relationship matrix and accounts for the inter-line relationships of the additive genetic effects. The non-additive genetic effects were assumed independent between lines.

The matrix $\boldsymbol{A}=\left\{ A_{jk} \right\}$ is defined as:

| $A_{jk}$=$\left\{ \begin{aligned} 1+F_{jn}, j=k \\ 2f_{jk}, j\neq k \end{aligned} \right.$ | (4) |
| --- | --- |

where $F_{jn}$ is the inbreeding coefficient of line$j$ adjusted to $n$ generations of sel-fertilization, and $f_{jk}$ is the coefficient of coancestry of line$j$ and $k$. The inverse of the $\boldsymbol{A}$ matrix was calculated from the pedigree data set following the iterative method described in (Meuwissen and Luo, 1992) but incorporating the adjustment of the inbreeding coefficient for the level of selfing (Oakey et al., 2006).

A parsimonious approximation of the $\boldsymbol{a}$ and $\boldsymbol{p}$ genetic effects was sought using the FA approach of Smith et al. (2001). With this approach the between trial variance-covariances of the $\boldsymbol{a}$ and $\boldsymbol{p}$ effects become:

| $\boldsymbol{G}_{a}=\boldsymbol{\Lambda}_{a}\boldsymbol{\Lambda}_{a}^{T}+\boldsymbol{\Psi}_{a}$  $\boldsymbol{G}_{p}=\boldsymbol{\Lambda}_{p}\boldsymbol{\Lambda}_{p}^{T}+\boldsymbol{\Psi}_{p}$ | (5) |
| --- | --- |

where $\boldsymbol{\Lambda}_{a}=\left[ \boldsymbol{\lambda}_{a_{1}}, \boldsymbol{\lambda}_{a_{1}}, \ldots\boldsymbol{\lambda}_{a_{k}} \right]$ and $\boldsymbol{\Lambda}_{p}=\left[ \boldsymbol{\lambda}_{p_{1}}, \boldsymbol{\lambda}_{p_{1}}, \ldots\boldsymbol{\lambda}_{p_{k^{'}}} \right]$ are matrices of loadings and $\boldsymbol{\Psi}_{a}$and $\boldsymbol{\Psi}_{p}$ are diagonal matrices of specific trial variances.

The joint distribution of the random effects $\left( \boldsymbol{a}, \boldsymbol{p}, \boldsymbol{u}, \boldsymbol{e} \right)$ was assumed to be Gaussian with zero mean and pairwise independent components. The variance covariance structures for $\boldsymbol{u}$ and $\boldsymbol{e}$ were identical to the structures defined for these effects in the models without pedigree.

**Factor analytic selection tools**

The predictions obtained of the additive effects were summarized using the factor analytic selection tools (FAST) derived in Smith and Cullis (2018), which provided measures of overall performance and stability for each line. These measures are based on the multiple regression representation of the additive genetic effects. Let ${\tilde{\boldsymbol{a}}}_{i}$ be the predicted additive genetic effects of the $i^{th}$ variety:

| ${\tilde{\boldsymbol{a}}}_{i}=\tilde{f}_{a_{1i}}^{*}{\hat{\boldsymbol{\lambda}}}_{a_{1}}^{*}+\tilde{f}_{a_{2i}}^{*}{\hat{\boldsymbol{\lambda}}}_{a_{2}}^{*}+\cdots{+\tilde{f}}_{a_{ki}}^{*}{\hat{\boldsymbol{\lambda}}}_{a_{k}+}^{*}{\tilde{\boldsymbol{\delta}}}_{ai}={\tilde{\boldsymbol{\beta}}}_{a_{i}}+{\tilde{\boldsymbol{\delta}}}_{a_{\boldsymbol{i}}}$ | (6) |
| --- | --- |

where ${\hat{\boldsymbol{\lambda}}}_{a_{1}}^{*}, {\hat{\boldsymbol{\lambda}}}_{a_{2}}^{*}, \cdots{\hat{\boldsymbol{\lambda}}}_{a_{k}}^{*}$ are the estimated loadings (Eqn. 5) rotated to a principal component solution for a meaningful interpretation and represent the independent variables of the regression, $\tilde{f}_{a_{1i}}^{*}, \tilde{f}_{a_{2i}}^{*}, \cdots{,\tilde{f}}_{a_{ki}}^{*}$ are the predicted rotated scores and represent the regression coefficients, and ${\tilde{\boldsymbol{\delta}}}_{a_{i}}$ is a lack of fit term. The FAST tools separate the common additive effects (${\tilde{\boldsymbol{\beta}}}_{a_{\boldsymbol{i}}}$) into the effects associated with the first factor and the rest, i.e. ${\tilde{\boldsymbol{\beta}}}_{a_{\boldsymbol{i}}}= \tilde{f}_{a_{1i}}^{*}{\hat{\boldsymbol{\lambda}}}_{a_{1}}^{*}+{\tilde{\boldsymbol{\epsilon}}}_{a_{i}}^{*}.$The overall performance (OP) is the fitted value at the mean of the first loading and the stability (Eqn. 7) is the root mean square deviation of (RMSD) from the regression line associated to the first loading.

| ${OP}_{i}=\tilde{f}_{a_{1i}}^{*}\bar{\lambda}_{1}=\frac{1}{t}\sum_{j=1}^{t} \hat{\lambda}_{a_{1j}}^{*}\tilde{f}_{a_{1i}}^{*}$ ${RMSD}_{i}=\sqrt{\frac{1}{t}\sum_{j=1}^{t} \tilde{\epsilon}_{a_{ij}}^{*2}}$ | (7) |
| --- | --- |

**Bibliography**

Meuwissen, T. H. E. & Luo, Z. (1992). Computing inbreeding coefficients in large populations. *Genetics Selection Evolution,* 24**,** 305-313, doi:10.1051/gse:19920402.

Oakey, H., Verbyla, A., Cullis, B., Wei, X. & Pitchford, W. (2007). Joint modeling of additive and non-additive (genetic line) effects in multi-environment trials. *Theoretical and Applied Genetics,* 114**,** 1319-1332, doi:10.1007/s00122-007-0515-3.

Oakey, H., Verbyla, A., Pitchford, W., Cullis, B. & Kuchel, H. (2006). Joint modeling of additive and non-additive genetic line effects in single field trials. *Theoretical and Applied Genetics,* 113**,** 809-819, doi:10.1007/s00122-006-0333-z.

Smith, A., Cullis, B. & Thompson, R. (2001). Analyzing variety by environment data using multiplicative mixed models and adjustments for spatial field trend. *Biometrics,* 57**,** 1138-1147, doi:10.1111/j.0006-341X.2001.01138.x.

Smith, A. B. & Cullis, B. R. (2018). Plant breeding selection tools built on factor analytic mixed models for multi-environment trial data. *Euphytica,* 214**,** 143-161, doi:10.1007/s10681-018-2220-5.
