# Supplementary material for: Incorporating the pedigree information in multi-environment trial analyses for improving common vetch
Source: Front Plant Sci. 2023 Aug 16;14:1166133. doi: 10.3389/fpls.2023.1166133 (PMC10467272; doi:10.3389/fpls.2023.1166133)
Supplement: Supplementary file 1 [file DataSheet_1.zip › Data Sheet 1/Supplementary material legends.DOCX]

Supplementary Material

**Incorporating the pedigree information in multi-environment trial analyses for improving common vetch**

Isabel Munoz Santa^*^, Stuart Nagel, Julian Daniel Taylor

*** Correspondence:** Isabel Munoz Santa: sabela.munozsanta@adelaide.edu.au

# Supplementary Tables, Figures and Appendix

## Supplementary Tables

**Supplementary Table S1**. REML estimates of the linear row and range slopes, block, spline in the row direction, row, range and residual variance, correlation in the range and row direction.

**Supplementary Table S2**. REML estimates of the additive, non-additive, and total genetic variance. Percentage of total genetic variance explained by the additive and non-additive component.

## Supplementary Figures

Supplementary Figure 1. Cluster diagram of the environments based on the estimate of the additive genetic correlation matrix. The line is used as a cut-off to define groups such that the average pairwise additive genetic correlation between groups do not exceed 0.5

Supplementary Figure 2. Cluster diagram of the environments based on the estimate of the non- additive genetic correlation matrix. The line is used as a cut-off to define groups such that the average pairwise non-additive genetic correlation between groups do not exceed 0.5

Supplementary Figure 3. Comparison of the empirical best linear unbiased prediction (BLUPs) of the total genetic effects for the standard versus pedigree multi-environment trial (MET) analysis in each experiment.

Supplementary Figure 4. Regression plots associated to the first loading for the varieties with the highest overall performance value. Magenta and blue points represent common additive genetic effects and differentiate whether the variety was present or not in the environment. The black solid points represent the overall performance values of the varieties.

## Supplementary Appendix

Supplementary Appendix I: Extended explanation of the statistical methodology
