# Supplementary figures and images for: Incorporating the pedigree information in multi-environment trial analyses for improving common vetch
Source: Front Plant Sci. 2023 Aug 16;14:1166133. doi: 10.3389/fpls.2023.1166133 (PMC10467272; doi:10.3389/fpls.2023.1166133)

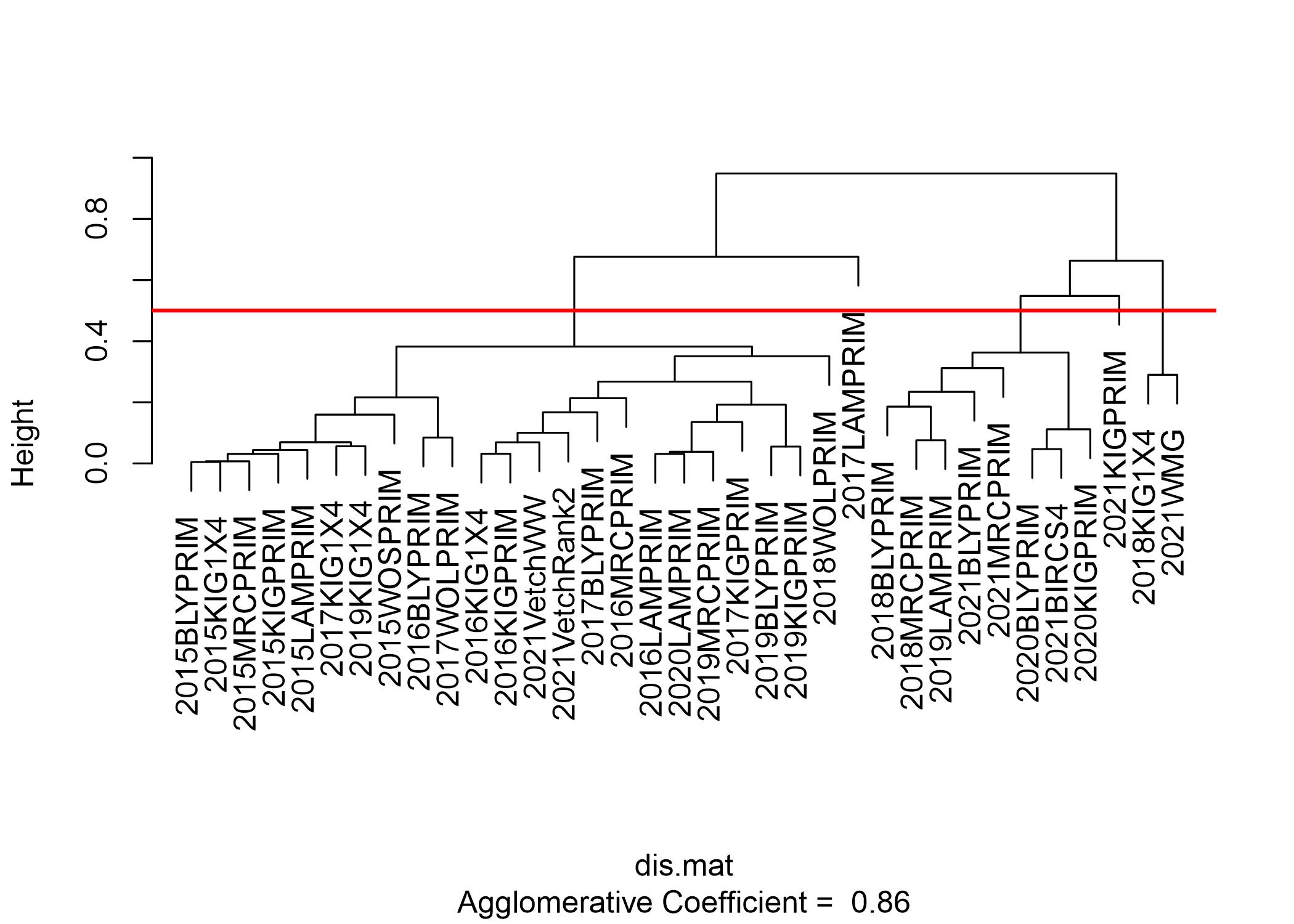

Supplement: Supplementary file 1 [file DataSheet_1.zip › Data Sheet 1/Image 1 (40).JPEG]

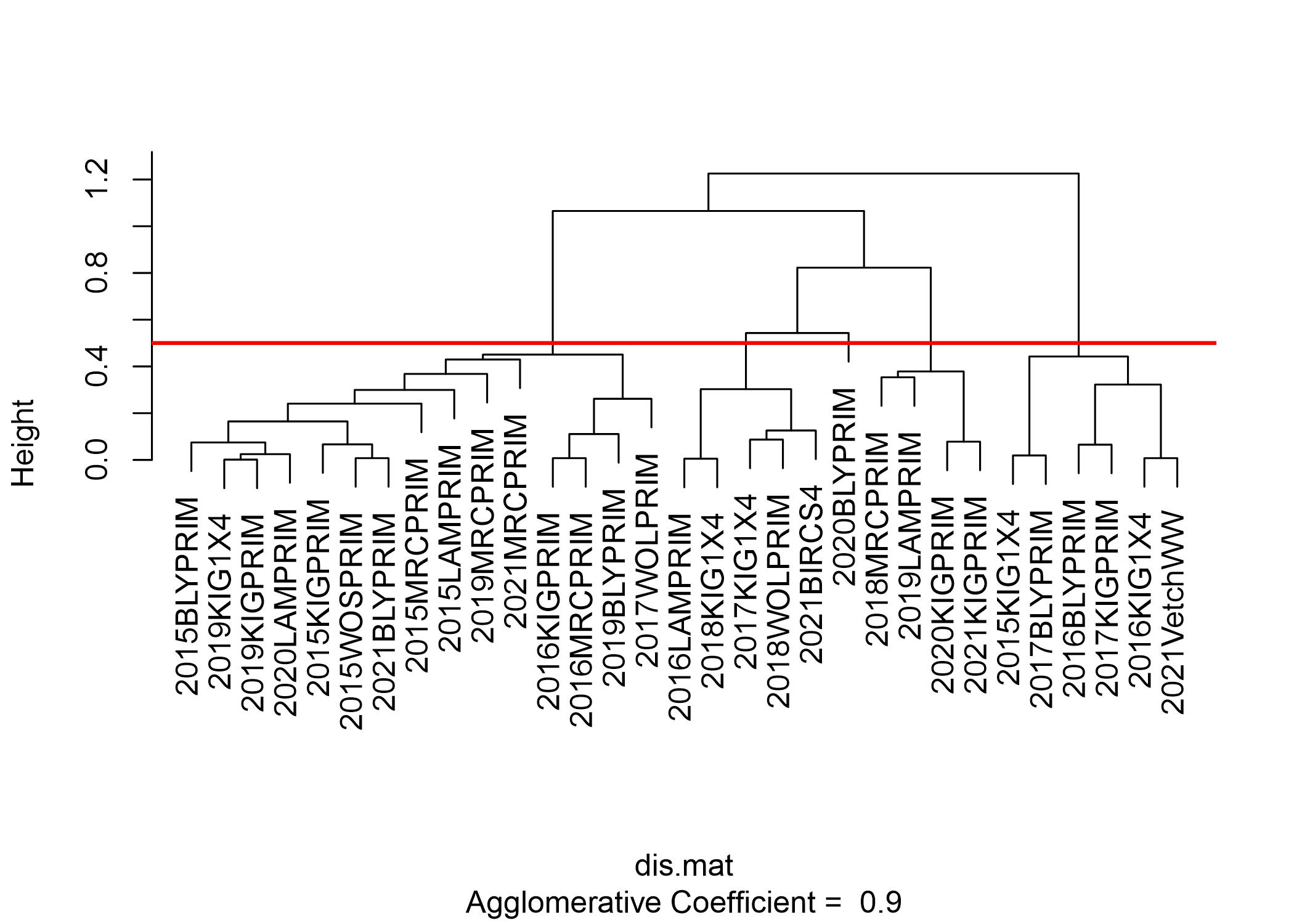

Supplement: Supplementary file 1 [file DataSheet_1.zip › Data Sheet 1/Image 2 (21).JPEG]

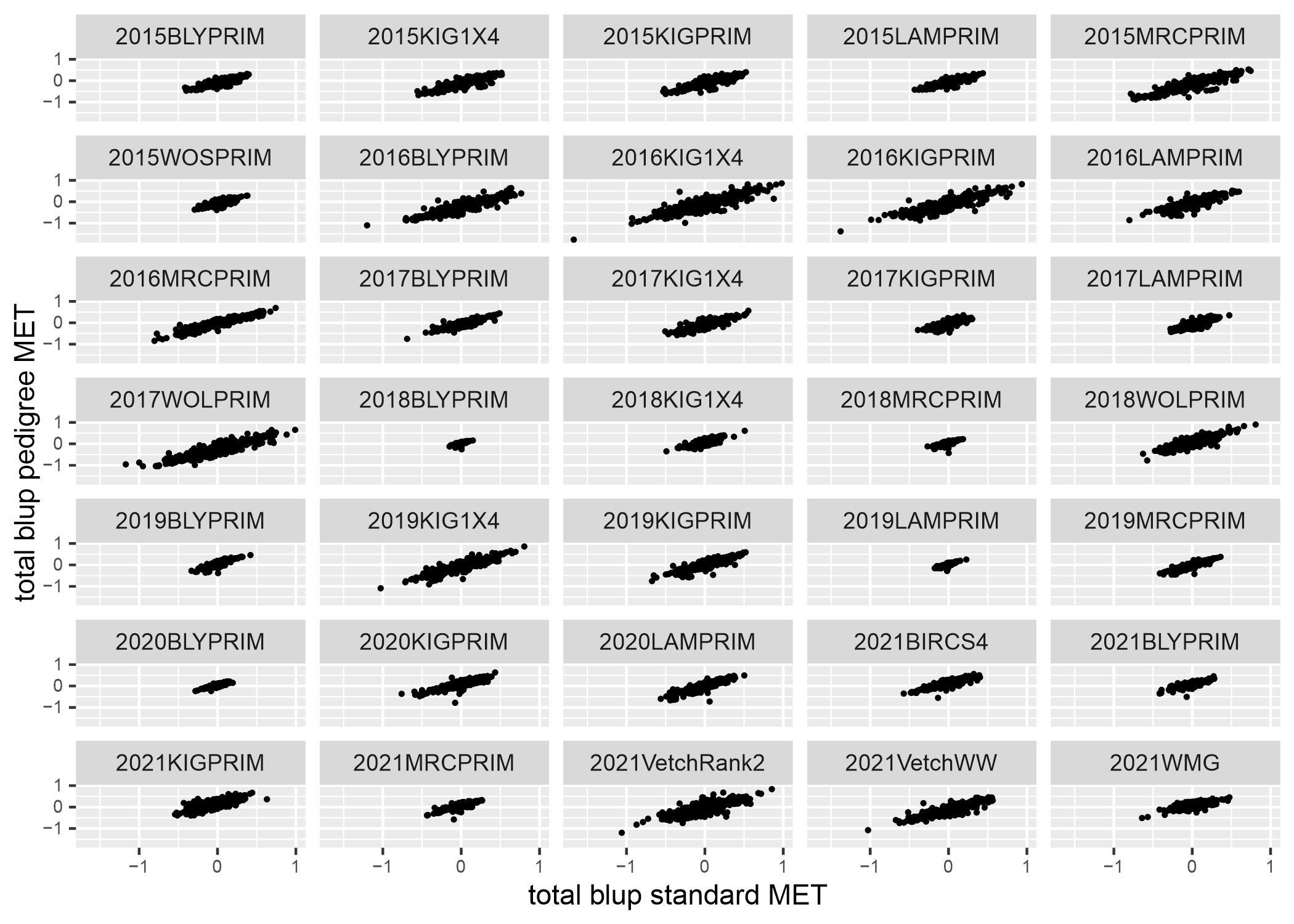

Supplement: Supplementary file 1 [file DataSheet_1.zip › Data Sheet 1/Image 3 (11).JPEG]

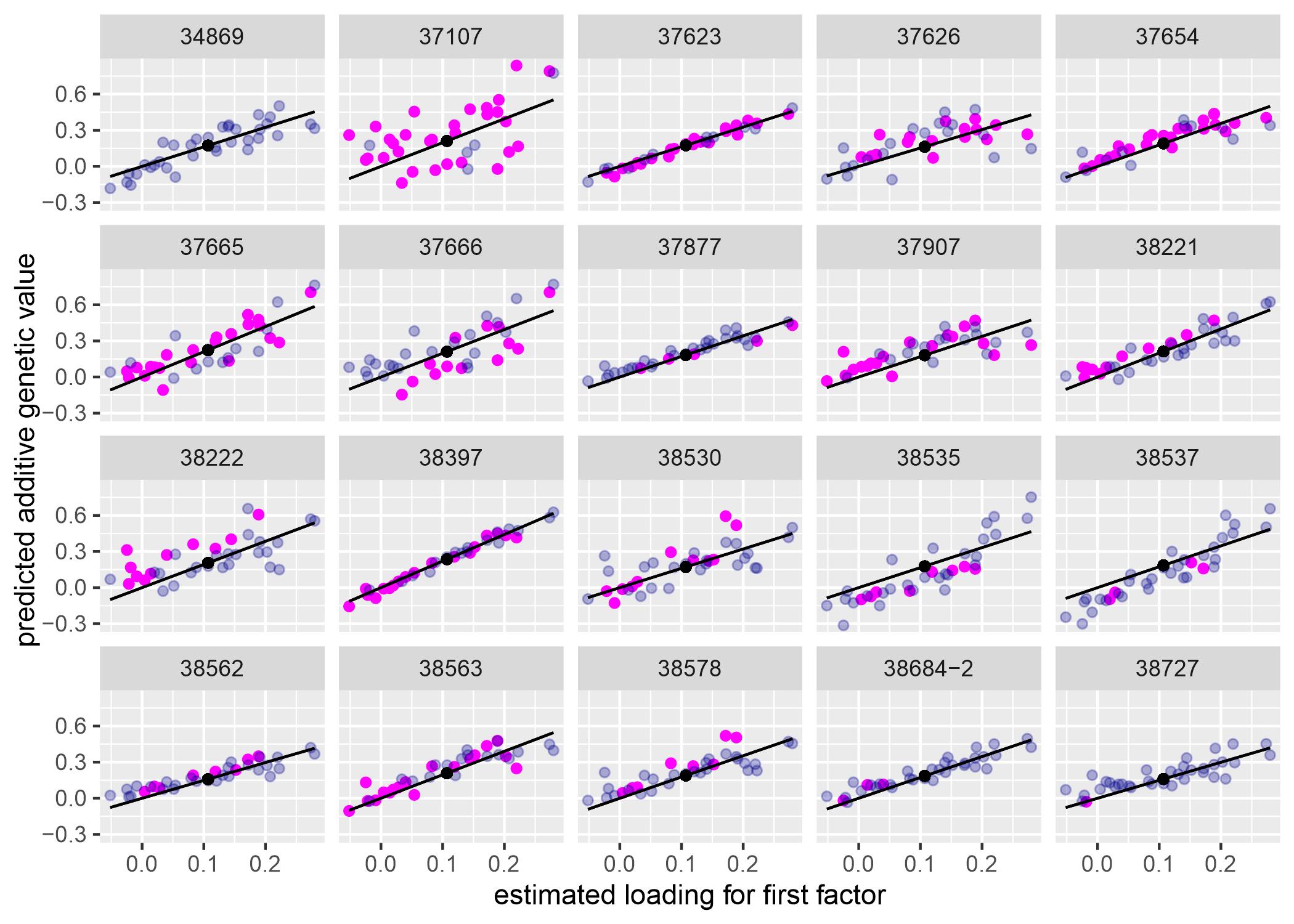

Supplement: Supplementary file 1 [file DataSheet_1.zip › Data Sheet 1/Image 4 (9).JPEG]
